# A dose and time response Markov model for the in-host dynamics of infection with intracellular bacteria following inhalation: with application to *Francisella tularensis*

**DOI:** 10.1098/rsif.2014.0119

**Published:** 2014-06-06

**Authors:** R. M. Wood, J. R. Egan, I. M. Hall

**Affiliations:** Bioterrorism and Emerging Disease Analysis, Microbial Risk Assessment and Behavioural Science, Public Health England, Porton Down SP4 0JG, UK

**Keywords:** systems biology, dose–response relationship, infectious disease incubation period, in-host mechanistic model, Markov process, *Francisella tularensis*

## Abstract

In a novel approach, the standard birth–death process is extended to incorporate a fundamental mechanism undergone by intracellular bacteria, phagocytosis. The model accounts for stochastic interaction between bacteria and cells of the immune system and heterogeneity in susceptibility to infection of individual hosts within a population. Model output is the dose–response relation and the dose-dependent distribution of time until response, where response is the onset of symptoms. The model is thereafter parametrized with respect to the highly virulent Schu S4 strain of *Francisella tularensis*, in the first such study to consider a biologically plausible mathematical model for early human infection with this bacterium. Results indicate a median infectious dose of about 23 organisms, which is higher than previously thought, and an average incubation period of between 3 and 7 days depending on dose. The distribution of incubation periods is right-skewed up to about 100 organisms and symmetric for larger doses. Moreover, there are some interesting parallels to the hypotheses of some of the classical dose–response models, such as independent action (single-hit model) and individual effective dose (probit model). The findings of this study support experimental evidence and postulations from other investigations that response is, in fact, influenced by both in-host and between-host variability.

## 1. Introduction

Following a covert release of hazardous biological material, whether naturally occurring or terrorist-related, there will be a need by public health authorities to characterize the extent of the hazard in order to minimize the number of casualties. This is especially the case if illness is severe and prophylactic medical countermeasures are needed to be deployed. Thus, it is critical to have an evidence-based assessment of the dose and time response relationship in individuals to understand the impact of a biological release on a population.

Mathematical models can be valuable tools in the analysis of dose and time response relationships, because there is typically insufficient experimental evidence to inform understanding across all doses of interest, potentially helping to characterize uncertainty in experimental results. However, the reliability of any estimation is critically dependent on the model used, and its suitability in representing the biological phenomena related to the pathogen in question. The earliest efforts were at the beginning of the twentieth century, and concern a hypothesis that response (i.e. illness or death) occurs in an individual only if the exposed dose is greater than an innate tolerance level. Irwin [1] refers to the least dose required for response as the *individual effective dose*. In order to appreciate the observed variability in host response for different levels of dose, the tolerance levels of individuals are assumed to be distributed throughout a population, typically by the lognormal distribution [1,2] (i.e. the commonly referred to probit model of dose–response). Other distributions can also be used—for example, the Weibull distribution [3].

Rather than assuming that hosts react differently to infection, the single-hit model [4] assumes that individuals are homogeneous, but the in-host biological mechanisms are stochastic, and not deterministic [5], processes. The hypothesis of the single-hit model is that bacteria act independently—*independent action*—and that each of the bacteria has a chance of alone invoking response (say *p*_hit_). For a dose of size *k,* the probability of response, *P*_R_, is therefore *P*_R_(*k*) = 1 − (1 − *p*_hit_)*^k^*. Thus, the dose–response relation can be recast as the exponential distribution function,1.1
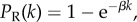
with *β* = −log(1 − *p*_hit_) (indeed *β* ≈ *p*_hit_ when *p*_hit_ is small). A crucial difference between independent action and individual effective dose is in the way in which the bacteria are perceived to interact; maximum synergism between bacteria in the latter (because the size of the dose alone dictates outcome), contrast with none in the former (because bacteria act independently).

Time until response can be incorporated within the exponential dose–response model by defining *β* in equation (1.1) as a function of time and not as a constant. Functions that have been used to this end are the distribution functions of the exponential, Weibull, lognormal and gamma distributions [6,7]. These distributions are commonly fitted to incubation period data for a broad spectrum of infectious diseases, such as AIDS [8], Creutzfeldt–Jakob disease [9], severe acute respiratory syndrome [10] and Legionnaires' disease [11]. For example, if the exponential distribution function is used, then1.2
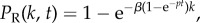
where *t* is the time post-exposure and *p* is the shape parameter.

This approach has been used to model infectious diseases caused by various intracellular bacteria (i.e. those that replicate within cells), including *Francisella tularensis*. In [7], the exponential dose–response relation (equation (1.1)) is fitted to monkey tularaemia data with the aforementioned distribution functions used for time until death. Although the authors state that their analysis serves to *model the in vivo bacterial kinetics*, there is a limited appreciation of the biological mechanisms at play, and the distributions are selected only on their ability to approximate the data. While the authors acknowledge the single-hit interpretation of the exponential dose–response relation, they do not discuss what might constitute a ‘hit’, or the appropriateness of a hit-type model for this bacterium.

A mechanistic interpretation of the exponential dose–response relation has been considered for anthrax. Brookmeyer *et al.* model the inhalation of (toxin-producing) *Bacillus anthacis* spores in humans under the assumption that the hit required to provoke illness constitutes a spore germinating before being cleared from the lung [6]. They therefore interpret *β* through the competing risks of spore clearance (rate *μ*) and germination (rate *λ*) such that *β* = *λ*/(*λ* + *μ*). It is assumed that the time from exposure to germination is exponentially distributed, giving rise to a relation equivalent to equation (1.2). But before symptoms occur, the germinated bacterium must multiply in order to produce toxins, the time for which is (also) assumed to be exponentially distributed. Thus, the final relation, *P*_R_(*k*, *t*), is given by the convolution of equation (1.2) and an exponential distribution.

**Figure 1.**
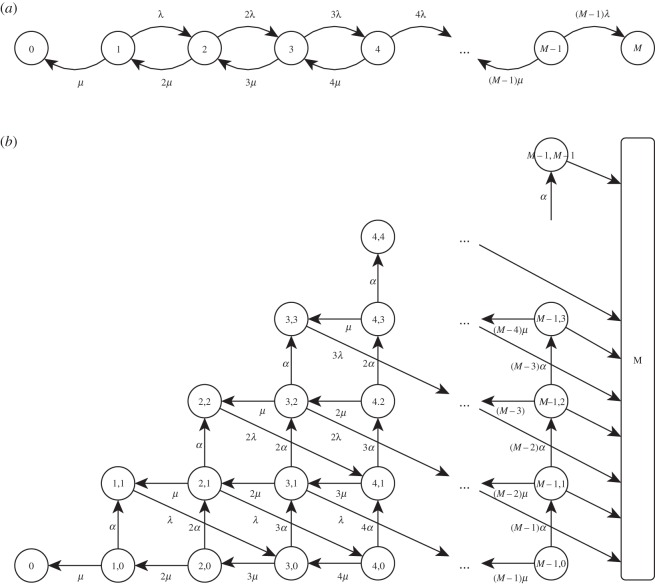
Depiction of the Markov chain of the (*a*) birth–death process with state definition {*B*} where *B*∈ℕ is the number of bacteria, and (*b*) birth–death–survival process with, as an example, *G* = 3 and state definition {*T*,*P*} where *T*∈ℕ is the total number of extracellular bacteria, *B*∈ℕ, and bacteria-containing phagocytes, *P*∈ℕ. The birth, death and survival rates are *λ* > 0 and *μ* > 0 and *α* > 0 respectively and the threshold for illness is *M*∈ℕ.

A different approach that also has an appreciation of biological mechanisms is introduced in [12]. Here, the number of bacteria grow exponentially at a given rate, *γ* > 0, until a threshold, *M*, is reached, at which point response is said to occur. If bacterial growth is due to bacterial division (at rate *λ* > 0) and death (at rate *μ* > 0), then *γ* = *λ* − *μ* can be interpreted as the net growth rate. This gives rise to the birth–death process—a special case of a continuous-time Markov chain. Here, the state of the system represents the number of bacteria, which can be increased by one through a birth (bacterium divides) and decreased by one through a death (bacterium killed). There is an absorbing barrier at zero (resolution of infection) and at the threshold, M∈ℕ (response). The Markov chain for this process is depicted in figure 1*a*.

A deterministic solution of the birth–death process for time until response is obtained by solving d*B*/d*t* = *γB* with *B*(0) = *k*, where *B*(*t*) is the number of bacteria at time *t*. This yields1.3
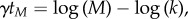
where *t_M_* is the (dose-dependent) time until response. Equation (1.3) is used in [13] to provide the mean to the lognormal distribution the authors use to represent the incubation period of inhalational tularaemia in humans, albeit with no biological interpretation (note that the authors also fit the exponential dose–response relation (equation (1.1)) in a separate approach). A stochastic solution to the birth–death process is presented in [14,15] (with Poisson-distributed initial dose). In both studies, it is assumed that 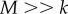, and results are provided for the dose–response relation (found to be exponential, i.e. equation (1.1)) and the dose-dependent distributions of time until response. A stochastic solution that does not make such an asymptotic assumption is provided in the electronic supplementary material, A, by means of a matrix-analytic approach.

While the birth–death process provides a mechanistic model capable of representing the stochasticity with respect to the in-host dynamics, it fails in providing a representation of the inherent variability relating to the heterogeneity of individuals. Furthermore, the birth–death process may be an appropriate model for bacteria that replicate extracellularly (such as *Streptococcus pyogenes* and *Escherichia coli* [16]) but it is not suitable for intracellular bacteria that reproduce within cells (such as *Bacillus anthracis*, *Legionella pneumophila* and *Salmonella enterica*).

In this study, the standard birth–death process is extended to take account of these limitations; incorporating host heterogeneity as well as the fundamental mechanism undergone by intracellular bacteria—phagocytosis. In particular, this model is concerned with non-toxin-producing obligate intracellular bacteria (which replicate solely within host cells) as opposed to facultative bacteria (which also reproduce in the extracellular environment). To illustrate the benefits of these inclusions in approximating dose–response and time until response, the model is applied to *F. tularensis* as an example.

This bacterium is selected for two reasons. First, it is of concern as a potential weapon of bioterrorism [17] and is the only non-toxin-producing obligate intracellular bacterium to appear on the Centers for Disease Control and Prevention list of category A bioagents [18]. Second, it has seen very little research with regard to dose–response modelling, with a literature search revealing only three such studies [3,7,13]. However, in none of these studies is a mechanistically derived model presented which appreciates the biological mechanisms at play. Furthermore, in [3], only dose–response is investigated with no consideration of time until response, and in [7] death, rather than illness, is considered and the model is parametrized for monkeys and not humans. The most severe form of the disease is pneumonic (or respiratory) tularaemia, which is caused by inhalation of aerosolized particles [19]. Of interest to this study is the Schu S4 strain [20] of the highly virulent *tularensis* subspecies [17,21]. While this intracellular organism is considered facultative *in vitro*, it is thought to be obligate *in vivo* [21,22].

## 2. Material and methods

### 2.1. The model

The following birth–death–survival process is assumed for a particular threshold, *M*∈ℕ. First, the host inhales a quantity of organisms all of which are transported to the extracellular space within the lung (forthwith referred to as the lung-space). Here, they are predated upon by cells of the immune system that can kill the bacteria directly (by degrading the membrane) or through a process called phagocytosis, whereby phagocytic cells (such as macrophages, monocytes, neutrophils and dendritic cells) engulf the bacteria. However, upon phagocytosis, obligate intracellular bacteria can evade the antimicrobial defences and reproduce within the phagocyte. Following this intracellular proliferation, a phagocyte dies, releasing its contents into the lung-space. Thus, three events are possible: death (killing of bacteria) with rate *μ* > 0, survival (phagocytosis of bacteria not resulting in bacterial death) with rate *α* > 0 and birth (release of *G*∈ℕ bacteria from a bacteria-containing phagocyte) with rate *λ* > 0. Response is said to occur when the number of extracellular bacteria in the lung-space reaches the threshold, *M*. Conversely, infection is said to have resolved when the number of extracellular bacteria and bacteria-containing phagocytes both reach zero.

This can be modelled as a continuous-time two-dimensional Markov chain; a stochastic process, *X* = {*X*(*t*); *t* ≥ 0}, in which the state of the system is the double *X* = {*T*,*P*}, where *T* = *B* + *P* denotes the total number of extracellular bacteria, *B*∈ℕ, and bacteria-containing phagocytes, *P*∈ℕ. The Markov chain for this process is depicted in figure 1*b* with, as an example, *G* = 3 bacteria released upon phagocyte death. Note that the initial state is {*k*, 0}.

A deterministic solution of this process is obtained by solving d*B*/d*t* = *λGP*−(*μ* + *α*)*B* and d*P*/d*t* = *αB*−*λP* with *B*(0) = *k* and *P*(0) = 0 which yields2.1
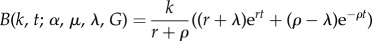
and2.2
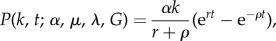
where 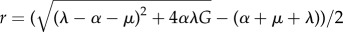 and *ρ* = *α* + *μ* + *λ* + *r*. Because *ρ* > 0, the second exponential term in equation (2.1) is always decaying. Thus, there can only be bacterial growth when *r* > 0, i.e. when *αG* > *μ* + *α*. Given a particular threshold, *M*, response occurs at time2.3
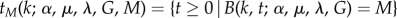
which, if the decaying term in equation (2.1) is small, has an asymptotic solution,2.4
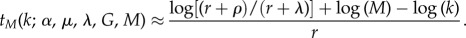
Note this is of a similar structure to that of the birth–death model (equation (1.3)).

**Figure 2.**
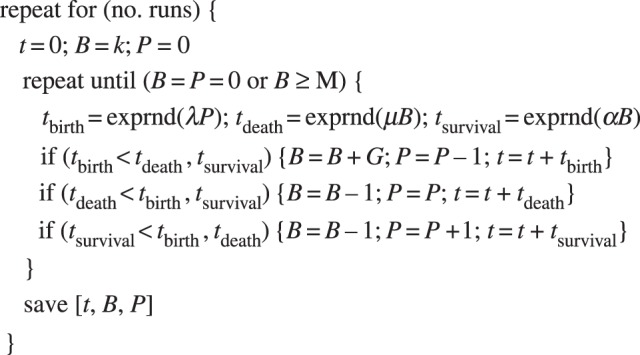
Pseudo-code to calculate the stochastic solution of the birth–death–survival model through the discrete-event simulation approach.

To obtain a stochastic solution, a matrix-analytic approach for the standard birth–death model is developed (the electronic supplementary material, A) and extended (the electronic supplementary material, B) yielding results for the probability of response and distribution of time until response for a particular threshold, *M*. In addition, a numerical solution is considered by means of discrete-event simulation (pseudo-code in figure 2). This approach serves to represent exactly the Markov model described above and provides equivalent results to the analytical approach (given, of course, a sufficient number of runs). An advantage of this is that host heterogeneity can be conveniently incorporated by sampling the threshold *M* from an appropriate distribution at each run of the simulation (an idea mentioned, but not adopted, in the birth–death model of [23] and in the simple bacterial growth model of [24]). Incorporating host heterogeneity (*individual effective dose*) alongside stochasticity with respect to bacteria–cell dynamics (*individual action*) has support from the *in vivo* studies [25,26], from which it is claimed that neither component of variability is alone sufficient to explain experimental results.

### 2.2. Parametrization

The birth–death–survival model is now parametrized for *F. tularensis*, with response characterized by the onset of symptoms. The derivation of estimates, summarized in table 1, is detailed as follows.

**Table 1.** Summary of parameter values.

parameter	notation	value	units
birth rate	*λ*	0.0241	per hour
number of bacteria released	*G*	358	organisms
death rate	*μ*	3	per hour
survival rate	*α*	0.0939	per hour
threshold	*M*	lognormal: *μ* = 26.2, *σ* = 6.05	log-hours

#### 2.2.1. Birth rate (*λ*)

**Figure 3.**
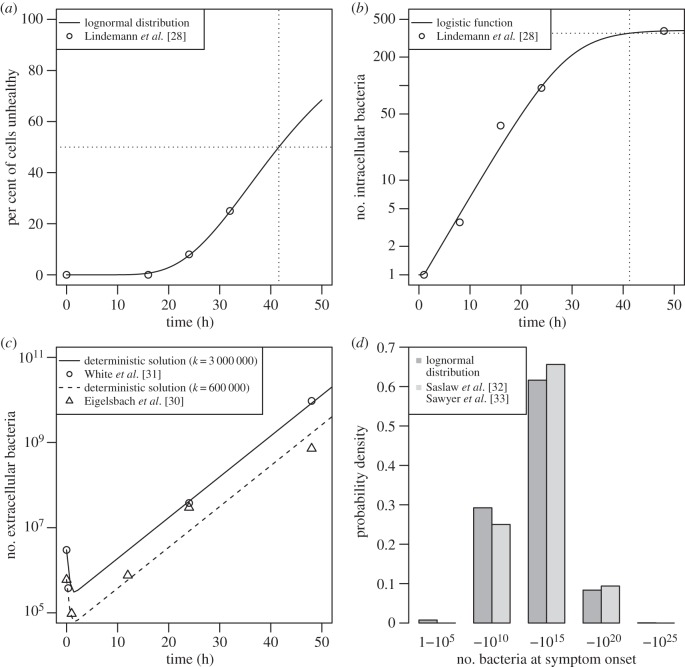
Parametrization, detailing (*a*) per cent of cells unhealthy over time, lognormal distribution function fitted to *in vitro* data of [28], (*b*) number of intracellular bacteria per cell over time, logistic function fitted to *in vitro* data of [28], (*c*) number of extracellular bacteria over time, deterministic solution for bacteria over time (equation (2.1)) fitted to *in vivo* data of [30,31], (*d*) number of extracellular bacteria present at illness onset, lognormal distribution fitted to estimations based on [32,33] using equation (2.1).

The reciprocal of the birth rate (i.e. rate of release of bacteria from phagocyte) is the mean time until bacterial release, which is associated with cell death [27]. In an *in vitro* study [28], human macrophages are infected with Schu S4. The authors find that at 16 h there is no evidence of cytopathogenicity and at 24 and 32 h, 8% and 25% of infected cells are unhealthy. Because cell death is governed by non-instantaneous processes [19], defining an actual time of death in such a study would not be possible. Here, the mean time until phagocyte death is taken as the time at which 50% of cells are unhealthy. However, such information is not expressly provided in [28], and so a value is estimated by fitting a distribution to the data that is available (by maximum likelihood). Because the final stages of the intracellular life cycle are poorly understood [29], the choice of distribution is not motivated by the biological mechanisms involved, but purely by goodness of fit. A lognormal distribution function (with log-mean 3.72 and log-standard deviation 0.385) is found to provide the best approximation to these data (by log-likelihood value) when evaluated alongside a gamma, Weibull and log–logistic distribution. With this distribution, a median time of 41.5 h (figure 3*a*) is found (2.5% of cells are unhealthy by 19.5 h and 97.5% unhealthy by 88.2 h); the reciprocal of which is the birth rate, i.e. *λ* = 0.0241 per hour.

#### 2.2.2 Number of bacteria released (*G*)

Lindemann *et al*. [28] measure the total number of Schu S4 bacteria, *b_*τ*_*, in cultured human macrophages at predefined times, 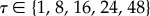 hours, post-infection. As it is assumed that each macrophage engulfs a single bacterium and that there is no growth within the first hour (because it takes 1 h for phagosomal escape [28]) then, on average, the number of bacteria per cell is 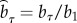. A logistic function is fitted to these data in order to capture the natural exponential growth of bacteria coupled with the stagnating effect of depleting nutrients. If *g*(*t*) is the total bacteria at time *t,* and *C* is the carrying capacity of the cell then, with the conditions *g*(1) = 1 and 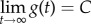,2.5
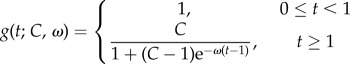
where *ω* is the growth parameter. Formula (2.5) (with *t* ≥ 1) is fitted to the data (figure 3*b*) by nonlinear least squares yielding *C* = 384 and *ω* = 0.212. The number of bacteria released upon cell death (at time *λ*^−1^) is calculated through equation (2.5) as *G* = *g*(*λ*^−1^) ≈ 358 (dotted line).

Because exponential growth occurs between *t_s_* = 1 h and *t_e_* = 24 h, then using the formula *g*(*t*) = *g*(1)·2*^t^*^/*d*^*,* the doubling time, *d*, is deduced as2.6
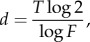
where *F* = *g*(*t_e_*)/*g*(*t_s_*) is the fold increase and *T* = *t_e_* − *t_s_*. Using equation (2.6), the intracellular doubling time is found to be 3.50 h.

#### 2.2.3 Survival rate (*α*) and death rate (*μ*)

These parameters cannot be directly deduced from the literature because they are dependent on a number of complex biological processes governed by the innate [34–36] and later, adaptive immune response [27,37–39]. Instead, they are estimated by fitting the formula for extracellular bacterial load over time of the deterministic birth–death–survival model (equation (2.1)) to *in vivo* data for infection with Schu S4 [30,31] (figure 3*c*). In these studies, monkeys are exposed to a high initial aerosol dose (600 000 and 3 000 000, respectively) and are sacrificed at predetermined times of up to 72 h. In both experiments, extensive morbidity (and in one case, mortality) is observed at 3 days, which is consistent with a short incubation period being associated with high challenge doses in monkeys exposed to type A *F. tularensis* [40]. To ensure that only the period of time until illness is considered, the result at 72 h is excluded for both studies. The model is fitted to the remaining data by nonlinear least squares (simultaneously, across both datasets) with a logarithmic transformation on the number of extracellular bacteria, such that log(*B*/*k*) = *θ* + *rt* + log(1 + e^−(*r* +^*^*ρ*^*^)*t* +^*^*φ*^*), where *θ* = log(*r* + *λ*) − log(*r* + *ρ*) and *φ* = log(*ρ* − *λ*) − log(*r* + *λ*). The corresponding estimators of survival rate and death rate are *α* = 0.0939 per hour and *μ* = 3 per hour, respectively. Note that the effective doubling time of the number of extracellular bacteria in the lung for the exponential growth phase is calculated as 3.47 h (cf*.* intracellular doubling time).

#### 2.2.4. Threshold (*M*)

The final step in the parametrization of the model is the determination of an appropriate distribution for the number of extracellular bacteria within the lung-space required for illness. Because this could not be expressly inferred from the literature, it is deduced by fitting a number of distributions to estimations of the extracellular bacterial load at the time of illness onset. These are obtained by using the deterministic solution of the birth–death–survival model for extracellular bacteria over time (equation (2.1)) with data from studies in which humans are exposed to aerosolized Schu S4, and inhaled dose and incubation period are explicitly recorded. To this end, the datasets of [32,33] are used, which each contain 16 subjects. The lognormal distribution (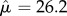, 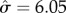 log-hours) is found to provide the best fit to these data (figure 3*d*) by maximum likelihood.

## 3. Results

The results are produced using the discrete-event simulation approach outlined in Material and methods. While an analytical solution has been formulated (see electronic supplementary material B), it is not used due to computational feasibility issues associated with the substantial threshold found for *F. tularensis*. The programming language R has been used in the computation of these results.

### 3.1. Dose–response

**Figure 4.**
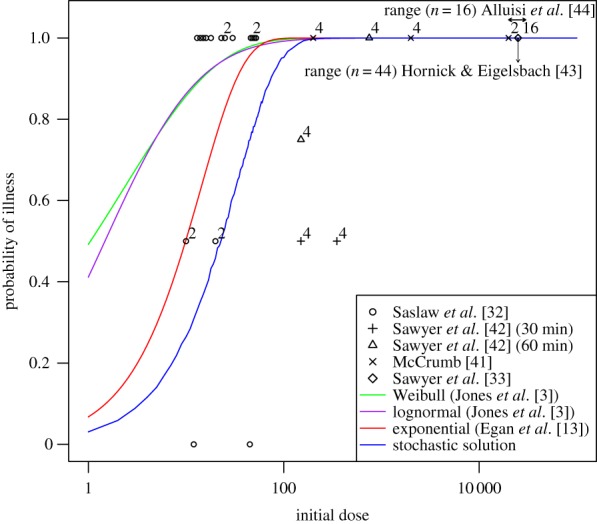
Dose–response relation of the stochastic solution of the model (blue line) and others obtained in the literature [3,13] for comparison. Data from a number of relevant studies [32,33,41–44] are also plotted (superscript numbers on points indicate number of subjects if greater than one). Arrows represent a range for studies in which an explicit result is not provided.

A median infectious dose of 22–23 organisms is deduced from the dose–response relation (figure 4 blue curve). This result is verified from the matrix-analytic approach (the electronic supplementary material, B), with *M* set equal to 500 (a value large enough such that response is inevitable if reached—i.e. a ‘point of no return’—see later). In order to assess the validity of the model, results are also plotted from a number of experimental studies involving human infection with aerosolized Schu S4 [32,33,41–44] (in [42], the inoculum is aged prior to delivery). Note that none of these data has been used within the parametrization of the model. Also included are a number of dose–response relations that have been produced in other studies (the dose–response relation derived in [7] is not included, because it considers the infection of monkeys and not humans).

Interestingly, the solution is well approximated by an exponential distribution (visually indistinguishable from the blue line in figure 4; not shown), indicating that the birth–death–survival model for *F. tularensis* may be interpreted as a single-hit-type model. To explore this further, consider the time immediately after exposure. Here, there are no bacteria-containing phagocytes and two events can occur: death (killing of bacteria), with probability *p*_d_ = *μ*/(*μ* + *α*) = 0.97, or survival (phagocytosis of bacteria not resulting in bacterial death), with probability *p*_s_ = *α*/(*μ* + *α*) = 0.03. If just one bacterium survives phagocytosis and proliferates then ultimately 358 bacteria are released back into the lung-space. To resolve infection, then all of these bacteria must be killed and this occurs with probability 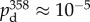 (as events are independent). Hence, the survival of just one bacterium is effectively sufficient to cause illness. This can be modelled by a binomial distribution (§1) which gives rise to the exponential distribution function (equation (1.1) with *β* ≈ *p*_s_). Therefore, the birth–death–survival model for *F. tularensis* can be interpreted as a single-hit-type model whereby a ‘hit’ is defined as the failed phagocytosis of a single bacterium. Note that if the threshold is fixed, say at the median *M*_med_ = 2.4 × 10^11^, then an equivalent dose–response relation is produced (because the probability of resolution is negligible for just 358 extracellular bacteria).

### 3.2. Time until response

**Figure 5.**
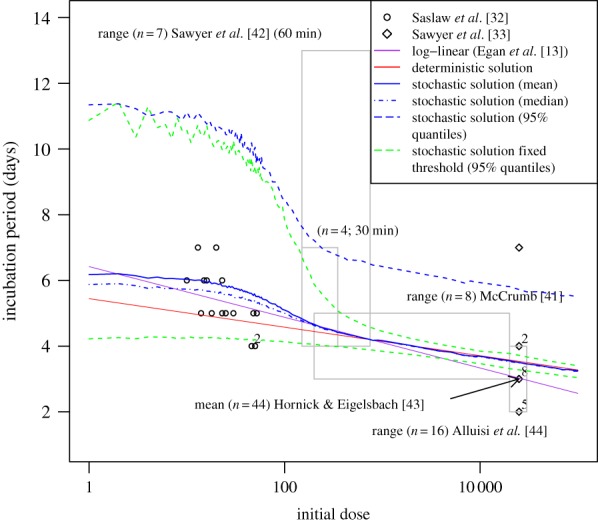
Time until response of the deterministic solution of the model (red line), the stochastic solution of the model (blue lines), the stochastic solution of the model with fixed threshold (green lines), and another obtained in the literature [13] for comparison. Data from a number of relevant studies [32,33,41–44] are also plotted (superscript numbers on points indicate number of subjects if greater than one). Light grey boxes represent a range for studies in which an explicit result is not provided.

The mean and median incubation period (i.e. time until illness) are plotted in figure 5 in addition to the 95% quantiles. For a coarse verification of these results, the deterministic solution, equation (2.3) with *M* = *M*_med_, is also included. As further verification, the median number of bacteria-containing phagocytes at the time of illness for the simulation results is assessed and found to be within an order of magnitude of the deterministic solution (equation (2.3) into equation (2.2)) at all doses. For comparison, data from a number of relevant studies involving human infection with aerosolized Schu S4 [32,33,41–44] are included within figure 5. Also included is the log-linear relation (equation (1.3)) obtained in [13] by fitting specifically to the data of [32,33]. This is equivalent to *fitting* the birth–death–survival deterministic solution (equation (2.3)) to these data (rather than using the parametrized solution), because when the decaying term in equation (2.1) is small the approximation given by equation (2.4) is also log-linear.

Results for the birth–death–survival model with a fixed threshold (set at the median, *M*_med_ = 2.4×10^11^) have also been deduced. The 95% quantiles (plotted) indicate a significant difference in dispersion, particularly at doses larger than 100 organisms. Note that the averages (mean and median) of this model are approximately similar to that with a distributed threshold (not shown).

**Figure 6.**
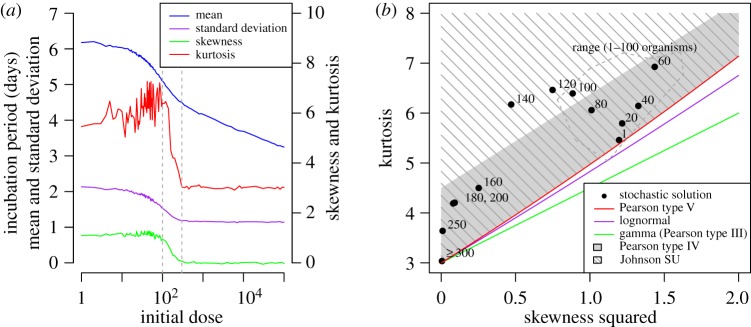
Time until response of the stochastic solution of the model. (*a*) Summary statistics (mean, standard deviation, skewness, kurtosis) with grey dotted lines indicating three dose ranges for which these measures display similar trends; (*b*) relationship between square of skewness and kurtosis (superscript numbers on points indicate initial dose; values for doses up to 100 organisms are contained within the range outlined by the grey dashed line). The relationship between these values for some common distributions is also plotted for comparison.

It would appear that the distribution of incubation period (for the model with a distributed threshold) varies significantly with dose. To gain a better understanding of this relationship, the mean, standard deviation, skewness and kurtosis are plotted in figure 6*a*. It would appear that the values of these measures follow a trend for doses fewer than 100 (decreasing mean, standard deviation; increasing skewness, kurtosis), between 100 and 300 (all measures decreasing), and greater than 300 (decreasing mean; other measures constant). Clearly, the incubation period is normally distributed for the latter range (skewness of zero and kurtosis of three) with a mean approximated by the deterministic solution (equation (2.4), i.e. *t_M_* = 5.44 − 0.189 log(*k*)) and a standard deviation of 1.15.

In order to assess the appropriateness of some common distributions (required by many applications, e.g. outbreak back-calculation tools [11,45–47]) in representing the mechanistically derived distribution at doses below 300 organisms, the relationship between skewness (squared) and kurtosis is compared between these distributions and the model results (figure 6*b*). The potential significance of these comparisons and of the other results presented in this section are considered in more detail in Discussion.

## 4. Discussion

### 4.1. The model

While the birth–death model is, in itself, inappropriate for representing intracellular bacteria (§1), it has provided a useful foundation for the birth–death–survival model considered here. In the 1960s, there was considerable academic interest in the mathematics of the simple birth–death model, involving stochastic differential equations [14,15] and generating functions [48]. However, very few experimental studies have actually made use of these results, despite a thorough account [23] crediting their ability in representing data for a variety of diseases. That paper [23] and methods therein are however not without their critics. It is claimed [5] that while *the overall picture provided by the basic birth–death model corresponds remarkably well to what is found in practice*, the underlying interpretations are flawed and there is no experimental evidence to suggest any form of stochastic mechanism in the infection dynamics. However, this is later refuted in an *in vivo* study [40] (in which monkeys are exposed to aerosolized type A *F. tularensis*), whose conclusions state that *organisms act independently of each other* and that *clinical and anatomic manifestations of the infection occur when the bacterial burden attains a given level*.

A number of potential limitations of the birth–death–survival model are now discussed. The underlying Markov structure of the model restricts the choice of statistical distribution in representing the occurrence of the three events—birth, death and survival—to those for which the Markov property holds. The simplest choice is the exponential distribution (in which events occur at random). Because of the memoryless property of this distribution, the length of time from bacterial uptake until phagocytic death is unknown, and so it is not possible to deduce the number of bacteria released on death (through equation (2.5)). Instead, an average number is used for all such instances—corresponding to the average time until phagocytic death (§2.2.1). But by removing this legitimate source of variability, this falsely increases the certainty of any model results. In fact, this relates to another issue—the suitability of the exponential distribution in the first place. For death and survival, this choice is justified, because the movement of bacteria [49] and phagocytes [50] has similar properties to that of a random walk. However, for the time from bacterial uptake to cell death and the associated release of bacteria (birth), a simple in-cell mechanistic model (based on a pure birth process with saturation of available nutrients) suggests that the variance is being overestimated by the exponential distribution. In other areas, however, the variability may well be underestimated, because the model fails to account for variability in other sources that can affect dispersion, such as bacterial age [42], diameter [7,51], agglutination, number retained and deposition site [23].

Another potential limitation is in the assumption that the rates of birth, death and survival are constant and independent of time. While the magnitude of these rates changes as the adaptive immune response becomes more involved (§2.2.3), the timing and extent to which this occurs are not known (owing to a lack of understanding and data regarding this transition). Instead, it is assumed that the length of time from exposure until response is insufficient for any discernible effect of the adaptive response to become apparent. However, if the adaptive response does come into effect before the time of illness, then this would, at first, reduce the growth rate of extracellular bacteria; decreasing the probability of response and increasing the incubation period of those that do respond (this is more likely to affect those with lower dose). If the effect of the adaptive immune response on the event rates could be quantified, then the discrete-event simulation approach used in this study could be modified with ease.

Third, the model assumes the single infection of cells—that is, a phagocyte may engulf only one bacterium—the validity of which is unknown. In the review of the literature for *F. tularensis*, no information could be found regarding the number of bacteria that can be simultaneously phagocytosed by any one cell *in vivo* (although it is thought that uptake is low *in vitro* [28,52]). Finally, it is assumed that there is an unlimited supply of phagocytes. This assumption is made, because the mechanisms that govern the number of viable phagocytes within the lung-space are complex and potentially unknown and, in any case, there are insufficient data to provide a parametrization.

### 4.2. Parametrization

As with many virulent organisms for which human trials are few and far between, there exist few relevant data, which makes parametrization a particularly onerous task. Here, the parameters are estimated in four stages (§2.2), starting with the birth rate. The reciprocal of this is estimated through the median time until cell death—found to be 41.2 h. The magnitude of this duration is supported by the only other similar *in vitro* study that could be found [53], in which at 24 h 100% of cells are still healthy (cf*.* 92% in [28]). Pooling data from other, related studies is not undertaken, because the timing of cell degradation is dependent on a number of factors, such as the strain [28,34], host species [53] and route of infection [19]. There is also a dependence on the type of phagocytic cell infected [22,27], which could affect model accuracy. This is because the parametrization of *λ* and *G* is based on an *in vitro* study [28] that only considers the macrophage, whereas *in vivo*, a number of cell types could be involved—although, the principal target of *F. tularensis* is indeed the macrophage [27].

The number of bacteria released is estimated by determining the amount of intracellular bacterial growth that begins from phagocytosis until cell death. Assuming logistic growth following an initial delay (to account for phagosomal escape), it is found that there are approximately 358 bacteria at the time of death. While there is no comparable study to validate this result specifically, the validity of the intracellular doubling time can be assessed. For the first 24 h, this is found to be 3.5 h, which is within the range of 3–8 h (obtained from reported fold increase using equation (2.6)) found in [54] (in which human cells are infected with Schu S4) for an equivalent timeframe. As would be expected, the doubling time (in humans) is less than with attenuated strains [52,55].

The death and survival rates are estimated by fitting the (otherwise parametrized) deterministic solution to the birth–death–survival model for extracellular bacteria over time (equation (2.1)) to data from two *in vivo* studies (in which monkeys are exposed to aerosolized Schu S4). This is the only part of the parametrization in which animal and not human data are used and, as stated in [56], *animal models must be carefully reviewed for applicability to humans, because of the inherent variability in host/micro-organism interaction*. Here, it has been assumed that the birth, death and survival rates are equivalent in humans and monkeys, but the threshold for illness is lower (10 times according to [24], owing to monkey body weight being one-tenth that of a human). This supports the shorter incubation periods for monkeys (48–72 h) when compared with humans, and as a result only data up to 48 h are used in this study (latter times could involve illness which may indicate the transition to the adaptive response—see above). Note that attempting to estimate all four parameters of equation (2.1) through these data provides a set of non-unique parameter values owing to an insufficient number of degrees of freedom.

In the final part of the parametrization, the distribution of individual response thresholds is determined by fitting to estimates of the number of extracellular bacteria at the time of illness onset from two human volunteer studies in which dose and incubation period are recorded. Because these are the only appropriate data, and that there are no *in vivo* studies that concern the number of extracellular bacteria on illness for humans, a validation of the resulting distribution is not possible. However, the values are reasonable and consistent with bacterial burdens found in monkey *in vivo* experiments [30,31].

### 4.3. Results

Before discussing the main results of this study, some of those deduced during the parametrization are firstly reviewed. In estimating the survival and death rate (§2.2.3), the deterministic solution for extracellular bacteria over time (equation (2.1)) is fitted to data from the two *in vivo* studies (figure 3*c*). These data clearly suggest an initial drop in the number of extracellular bacteria followed by exponential growth (the rate of which is lower in less virulent strains [7]). A birth–death model cannot represent these characteristics (not shown, but exemplified in [23] in fitting to guinea pig plague data), but an accurate portrayal is obtained by extending this standard approach to incorporate phagocytosis. Furthermore, fitting the birth–death deterministic solution only yields the net growth rate, *λ* − *μ*, which does not provide an explicit parametrization required to obtain the dose–response relation and incubation period distribution (by either the asymptotic approaches of [14] and [15], or by the matrix-analytic approach detailed in the electronic supplementary material, A).

It is assumed that clinical infection occurs when some threshold number of bacteria is in the system. The triggering of symptoms is a very complex process with few data available to validate more detailed models. It is likely that the threshold will vary by individual, and it appears that a fixed threshold poorly describes the data (figure 5) at higher doses given the variability assumed in the other parameters. This is because the process is more deterministic in the higher dose range—an observation consistent with the standard birth–death model with host homogeneity [5,23]. On the other hand, if the threshold is lognormally distributed then, given deterministic exponential growth, the times at which the number of extracellular bacteria reach this threshold are normally distributed, as deduced for doses of 300 organisms and greater (not possible to verify from [43] owing to the recording sensitivity). This increased dispersion enables the model to capture the variability exhibited in the data for such doses. However, at lower doses, the majority of the variability is attributable to the stochastic in-host processes, so the characterization of the threshold itself plays a lesser role. This variability is caused by an initial bistability, but as the numbers of extracellular bacteria start to increase they reach a ‘point of no return’, from which the process becomes deterministic. This is supported in fitting the three-parameter lognormal distribution to low dose fixed threshold data, whereby an estimated location parameter of 3–4 days indicates the duration of the deterministic processes (cf*.* anthrax model of [47]). For the same dose range with the distributed threshold model, this and some other standard two-parameter distributions are unable to represent the skewness and kurtosis of the modelled incubation period distributions (figure 6*b*). In fact, the only distribution, out of those considered, that is able to do so is the (four parameter) Johnson SU family of distributions. While the gamma distribution would appear to provide the least appropriate choice, the lognormal distribution—long associated with modelling incubation periods [57,58]—is also clearly unsuitable.

In summary, an extension to the standard birth–death process has allowed for the incorporation of phagocytosis—a fundamental mechanism undergone by intracellular bacteria. Also considered is the heterogeneity of individuals through a distributed threshold required for illness. For *F. tularensis*, this raises some interesting parallels to some of the classical dose–response models. First, the infection dynamics for dose–response can be simplified to the single-hit model, whereby the hit required for response is a failed phagocytosis. Second, the thresholds are found to be lognormally distributed, as in the probit model. These findings thus support the experimental evidence and postulations of [25,26] that response is a mixture of both the hypotheses of independent action (single-hit model) and individual effective dose (probit model). Future uses of the model considered here could include application to *Coxiella burnetii* (Q-fever) and *L. pneumophila* (Legionnaires' disease).
